# SpatialPPI: Three-dimensional space protein-protein interaction prediction with AlphaFold Multimer

**DOI:** 10.1016/j.csbj.2024.03.009

**Published:** 2024-03-15

**Authors:** Wenxing Hu, Masahito Ohue

**Affiliations:** Department of Computer Science, School of Computing, Tokyo Institute of Technology, Yokohama, Kanagawa 226–8501, Japan

**Keywords:** Protein-protein interaction, Machine Learning, Convolutional Neural Network, AlphaFold

## Abstract

Rapid advancements in protein sequencing technology have resulted in gaps between proteins with identified sequences and those with mapped structures. Although sequence-based predictions offer insights, they can be incomplete due to the absence of structural details. Conversely, structure-based methods face challenges with respect to newly sequenced proteins. The AlphaFold Multimer has remarkable accuracy in predicting the structure of protein complexes. However, it cannot distinguish whether the input protein sequences can interact. Nonetheless, by analyzing the information in the models predicted by the AlphaFold Multimer, we propose a highly accurate method for predicting protein interactions. This study focuses on the use of deep neural networks, specifically to analyze protein complex structures predicted by the AlphaFold Multimer. By transforming atomic coordinates and utilizing sophisticated image-processing techniques, vital 3D structural details were extracted from protein complexes. Recognizing the significance of evaluating residue distances in protein interactions, this study leveraged image recognition approaches by integrating Densely Connected Convolutional Networks (DenseNet) and Deep Residual Network (ResNet) within 3D convolutional networks for protein 3D structure analysis. When benchmarked against leading protein-protein interaction prediction methods, such as SpeedPPI, D-script, DeepTrio, and PEPPI, our proposed method, named SpatialPPI, exhibited notable efficacy, emphasizing the promising role of 3D spatial processing in advancing the realm of structural biology. The SpatialPPI code is available at: https://github.com/ohuelab/SpatialPPI.

## Highlights

• SpatialPPI predicts protein-protein interactions based on the structure of protein complexes from AlphaFold Multimer results.
• SpatialPPI renders the predicted structures into 3D tensors through spatial-based rendering and analysis using deep neural networks.
• Achieves superior prediction performance of protein-protein interactions over other approaches.
• SpatialPPI boosts pathway analysis and drug target identification through accurate protein-protein interaction predictions.

## Graphical Abstract

**Figure:**
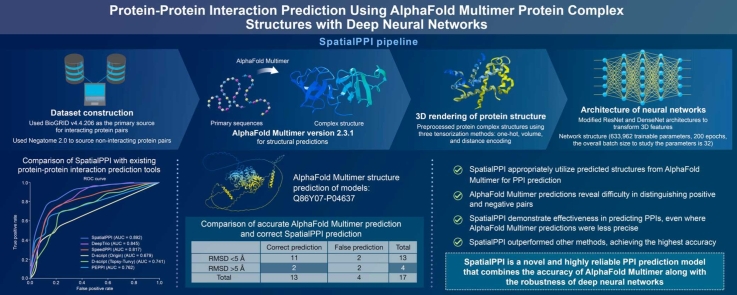

## 1 Introduction

Proteins are essential for various biological functions and form the basis of life by catalyzing reactions, transporting molecules, and forming cellular and tissue structures. Predicting protein-protein interactions (PPIs) is complex due to their intricate and dynamic interplay. Accurate PPI prediction aids in understanding underlying biological mechanisms and developing disease treatments. Currently, the dominant approaches for PPIs forecasting include sequence-based predictions [1], [2], [3], [4], which use the amino acid chains of two proteins as the input, and structure-based predictions [5], [6], which use experimentally determined 3D structures. PPI predictions can be categorized into similarity methods based on homologous interactions [7], [8] and machine learning-based methods [9], [10], [11], [12]. D-script [13] is currently one of the most widely applied machine learning-based prediction methods for directly predicting PPIs from protein sequences. D-script achieves this by utilizing a natural language processing approach to design a pretrained language model for generating input representations of protein sequences. Further, the D-script estimates the contact map of the protein and uses an interaction module to summarize the interactions. Another novel approach to predict PPIs is DeepTrio [14], which predicts PPIs by inputting protein sequences into a Siamese architecture with mask multiple parallel CNNs. It also provides visualization of the importance of each protein residue using both online and offline tools, along with additional predictions for single proteins. PEPPI [15] combines a homologous search and multilayer perceptron classifier [16] using Gaussian kernel density estimation. PEPPI uses a multi-prong pipeline to predict PPIs using a Gaussian kernel density estimation. These modules include a database lookup module, a conjoint triad-trained neural network, and two “interology” based modules: a threading-based module using a modified version of SPRING [17] and a sequence-based module using BLAST [18].

On the other hand, the exponential growth rate of known protein sequences promoted by the rapid development of automated sequencing technologies has led to a significant disparity in the pace of protein sequences and experimental protein structure determination. This trend has been widely observed [19] and is primarily due to the inherent complexity and time-consuming nature of the experimental methods used to determine protein structures. Alternatively, determining the protein sequence is relatively straightforward and can be achieved using automated sequencing technologies. An exponential sequence-structure gap was observed between the number of known structures in the Data Bank [20] and the number of known sequences in the UniProt database [21]. However, predicting PPIs based on the 3D structure of proteins is advantageous over sequence-based prediction as PPIs are frequently mediated by specific structural features such as complementary binding surfaces and specific hydrogen bonds, which can be more accurately modeled using 3D structure information than sequence information alone [22]. In contrast, sequence-based predictions of PPIs rely on identifying conserved motifs and domains involved in protein interactions, which can be disadvantageous due to the diversity of potential binding partners and the limited information provided by the sequence alone [23]. Furthermore, 3D structure-based predictions of PPIs offer a more comprehensive view of the interactions, including the types of interactions, such as hydrophobic and electrostatic interactions, and the specific residues involved [24]. Such detailed information not only provides insights into the mechanisms underlying PPIs but also significantly enhances pathway analysis, informing drug discovery efforts and the design of novel therapeutics. Overall, the use of 3D structural information for PPI prediction holds great promise for advancing our understanding of complex biological processes and developing new treatments for diseases. However, due to the complexity associated with obtaining protein structure information, the prediction methods based on this type of information face data insufficiency challenges and difficulty in predicting new proteins due to a lack of experimental data.

The AlphaFold Multimer [25] is a potential solution to the deficiency of structure-based PPI prediction methods. The AlphaFold Multimer is a deep neural network-based method developed by the AlphaFold team at DeepMind to predict the structure of protein complexes composed of multiple interacting proteins; thus, offering a novel approach for investigating PPIs. Due to the complex nature of PPIs, predicting their structures and functions remains a challenge in the field of structural biology. However, the ability of AlphaFold Multimer to generate accurate and reliable predictions of protein complex structures could significantly enhance our understanding of the mechanisms underlying these interactions. With the increased accuracy of structural predictions, insight can be gained into the binding sites and key residues involved in PPIs; thereby improving the ability to develop deep learning methods that predict these interactions.

The FoldDock algorithm [26] took advantage of protein complexes prediction methods. The FoldDock utilizes multiple sequence alignments (MSAs) generated using AlphaFold to generate pDockQ scores. The pDockQ score of FoldDock was calculated using a sigmoidal fit of the average pLDDT score [27] generated using AlphaFold. Thus, the FoldDock algorithm can predict the potential of two proteins to interact with each other and has achieved state-of-the-art accuracy. SpeedPPI is a PPI prediction method based on predicted protein structures. According to a description by the FoldDock team, SpeedPPI [28] is an improved version of FoldDock with enhanced usability and speed [29].

Furthermore, Jones et al. [30] have proposed an approach that utilizes a neural network for transforming 3D protein-ligand complexes into a 3D data structure by rescaling atomic coordinates at a 1 Å resolution to forecast protein-ligand binding affinity, which discussed a method for inputting protein structures to convolutional neural networks. In addition to these methods, 3D convolutional neural networks (3D-CNNs) have been extensively used in computer vision domains such as medical diagnosis for 3D image segmentation [31], [32] as well as recognizing gestures and activities in videos [33], [34]. Specifically, Densely Connected Convolutional Networks (DenseNet) [35] and Deep Residual Network (ResNet) [36] have become the main backbones of computer vision networks [37], [38]. Both network structures are designed to solve the degradation problem that occurs when the depth of the network structure increases; that is, the accuracy of the network saturates or even decreases as the depth of the network increases. Protein structure analysis is similar in this regard with computer vision data structure analysis. The image information is represented as a 3D array in computing, where the first two dimensions represent the spatial coordinates, and the third dimension represents the color information of the position. Furthermore, the techniques used for video analysis are similar to those used for protein processing. By stacking multiple images on top of each other, the first dimension represents time, the second and third dimensions represent spatial coordinates, and the last dimension represents the color information at that position. In deep learning, the core issues of computer vision and protein structure analysis are similar: finding regions with features by calculating the correlations between adjacent units in space, detecting patterns in these regions, and classifying them based on their features. Because of their similarities, ResNet and DenseNet are expected to be leverage for protein analysis [39], [40]. Together, these approaches provide a promising path for understanding the complex interactions between proteins and improving the ability to predict and model their behavior.

Therefore, we propose SpatialPPI, a method that uses deep neural networks to analyze protein complexes predicted by the AlphaFold Multimer to forecast PPIs. Spatial models of the protein complexes were determined by transforming the atomic coordinates and calculating the atomic distributions. This approach employs advanced image processing strategies to extract crucial 3D structural information from protein complexes. In this project, both DenseNet and ResNet backbone structures were implemented in 3D convolutional networks to resolve protein 3D structure data. The proposed method shows promising results in predicting PPIs, thereby highlighting the potential of 3D space-rendering processing methods in advancing structural biology research. The SpatialPPI code is available at https://github.com/ohuelab/SpatialPPI under the Apache-2.0 license.

## 2 Materials and methods

**Fig. 1:**
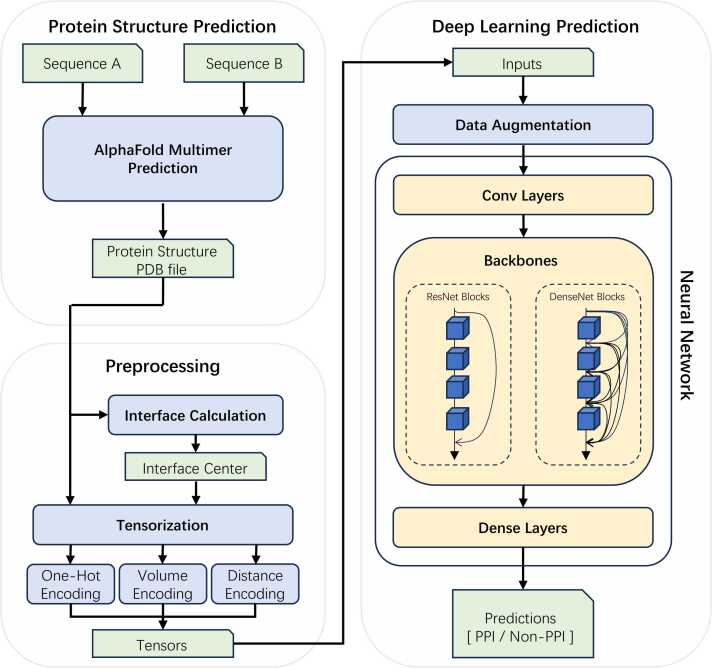
Flow chart of SpatialPPI. First, the AlphaFold Multimer predicts the two-input protein sequences and generates the structure of the protein complex, which is stored in a PDB file. The protein complex is tensorized by calculating its interface using either one-hot encoding, volume encoding, or distance encoding. The tensor is then passed to the neural network as input data. After data augmentation, the DenseNet backbone or ResNet backbone predicts and outputs the probability that the input proteins can interact with each other.

SpatialPPI is a pipeline that can predict the possibility of two single-chain proteins to interact with each other. The sequence information of the two proteins was used as the input to the pipeline. The structural information of the potential complex was predicted using the AlphaFold Multimer, and the prediction result was rendered into 3D tensors as the input to the convolutional neural network to generate the prediction. Fig. 1 shows the flowchart of SpatialPPI.

### 2.1 Dataset construction

The quality and reliability of PPI data are paramount for the development and evaluation of computational models and algorithms for PPI prediction. In this study, we used BioGRID v4.4.206 [41], a comprehensive biomedical interaction repository, as the primary source of interacting protein pairs. BioGRID contains more than 2 million experimentally validated PPIs from more than 70,000 publications in primary literature. This ensured the authenticity and accuracy of the positive datasets used in this study.

However, obtaining reliable negative datasets for PPI predictions remains challenging. As demonstrated by Wei *et al.* [42], experimentally validated datasets of non-interacting proteins are more reliable as negative datasets than randomly matched proteins that are not present in the positive set. To address this issue, we used Negatome 2.0 [43], a manually annotated literature data resource, to source non-interacting protein pairs for our study. Negatome describes the lack of protein interactions and includes 2171 non-interacting pairs from 1828 proteins.

Although both Negatome 2.0 and BioGRID are experimentally determined datasets, it should be noted that conflicts exist between the positive and negative datasets. A total of 26.5% of the pairs in the Negatome had conflicting records in the BioGRID, of which 296 conflicts had more than one publication in the BioGRID. To mitigate the detrimental effects of conflict on the integrity of the resulting dataset and enhance its overall quality, we engaged in a meticulous curation process. This process involved eliminating conflicts, duplicates, and self-interactions from the dataset. Additionally, when dealing with proteins having similar interactions with other proteins with high sequence identity or when two protein pairs had similar chains and the same interaction type (PPI/Non-PPI), we removed the pair which the interactor has fewer interactions to prevent data redundancy. This was done to circumvent the potential issue of sequences with substantial similarity in both training and test sets, which could lead to overfitting. Such overfitting adversely affects the evaluation outcomes, thereby not accurately representing the true efficacy of the model.

Furthermore, to ensure a balanced and diverse composition of the dataset, we implemented a criteria mandating that each protein be represented by at least one pair of interacting proteins and one pair of non-interacting proteins. This approach was adopted to maintain a comprehensive and representative dataset, essential for robust and reliable model training and evaluation. The final dataset comprises a total of 1200 protein pairs, consisting of 600 positive and 600 negative instances, derived from 375 *Homo sapiens* proteins. Overall, the resulting dataset represents a high-quality and reliable resource for the development and evaluation of computational models and algorithms for predicting PPIs.

### 2.2 Protein structure using AlphaFold Multimer

In view of the achievement of AlphaFold [44] in the 14th Critical Assessment of Structural Prediction Competition [45], the algorithm was used in this study to generate possible protein complexes of the input sequences. The AlphaFold Multimer uses the AlphaFold model to predict the structures of individual protein subunits and then assembles them into a complex using an optimization algorithm. The optimization algorithm considers both the predicted structure and binding affinity of each subunit to minimize the energy of the complex. This high-accuracy protein structure prediction method allows the pipeline to accept easy-to-acquire protein sequence data and simultaneously obtain more spatial information from the protein structure to improve the prediction accuracy.

The structural prediction of 1200 protein complexes was performed using AlphaFold Multimer version 2.3.1, which was released on January 12, 2023 [46]. The AlphaFold Multimer was executed using the jackhmmer search from HMMER3 [47] targeting Uniref90 [48], UniProt [49], and MGnify [50], and a joint search of the Big Fantastic Database [51], [52] and UniRef30[53] using HHBlits [54]. For each input, five different models were generated using the AlphaFold Multimer, and each model donated one prediction, yielding a total of 6000 Protein Data Bank (PDB) files.

### 2.3 3D rendering of protein structure

Extracting the features of input data and converting them into data structures that can be recognized by neural networks has always been an issue that has attracted the attention of deep learning researchers. The prediction results of the AlphaFold Multimer are in PDB format [55], which is a list of coordinates for atoms. The method used in this study is to map the preprocessed protein complex structure into a 3D tensor according to the coordinates. This method has also been used in previous studies [30], [56]. Its advantage is that, compared with linear data stored in PDB format files or two-dimensional data such as distance matrices, it can better express the spatial structure information of proteins; thus, providing more information for the input part of the neural network. In addition, it allows for drawing conclusions that may not have been summarized by human analyses. The current opinion is that whether proteins can bind to each other depends mainly on the contact surfaces of the two proteins [57]. Therefore, in this study, the contact surface of the protein complex was selected as the center of the input data. To this end, the preprocessing stage consists of removing the predicted low-confidence parts, calculating the residues belonging to the contact surface, and building a tensor centered on the geometric center of the contact surface.

**Fig. 2:**
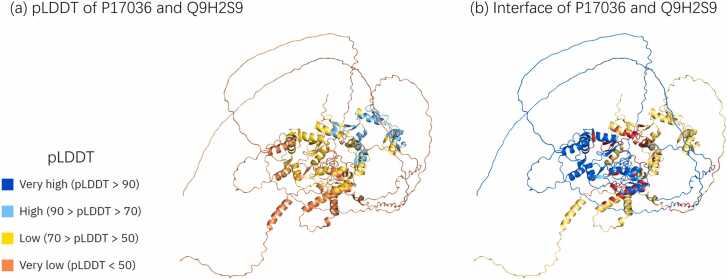
Examples of disordered areas, which usually appear in the AlphaFold prediction result. Disordered areas are often flexible and float in space. In the prediction results of the AlphaFold Multimer, the disordered area is often intertwined with the rest of the structure, and the distance is lower than the common contact threshold, which is 8 Å. (a) and (b) Represent AlphaFold Multimer predicted structure of protein complexes P17036 and Q9H2S9. (a) The pLDDT score of the predicted structure, where the color indicates the level of the pLDDT score. It can be observed that there is a large disordered area on the periphery of the structure. (b) The interface between the two structures. The blue and yellow parts represent two chains, respectively, and the red area represents the interface residues whose distance is less than 8 Å. Multiple contact areas formed by disordered areas intertwining with each other are presented in the figure.

Disordered structures are common in eukaryotic proteomes. Previous studies have estimated the proportion of disordered residues in the human proteome to range between 37–50% [58]. The AlphaFold research team discovered that the pLDDT is an adequate predictor of intrinsically disordered areas because the distribution of pLDDT between resolved and unresolved residues in the PDB sequence is substantially dissimilar [53]. The same study indicates that AlphaFold places long domains with pLDDT scores of less than 50 to exhibit a ribbon-like appearance and should be interpreted as a prediction of a disordered state and not as structural information [59]. Hence, these parts had no substantial effect on the structure of the protein interactions. However, these free, long, single chains are often entangled with another chain to form a part of the region where the two proteins are proximal to each other (Fig. 2). Therefore, long single strands with pLDDT scores > 50 were deleted to reduce the influence of contact regions other than the interface predicted by AlphaFold.

The center of the tensor was defined as the geometric center of the residue in contact with the two chains. The interface calculation was primarily performed by calculating the distance matrix of carbon atoms using the remoteness indicator code alpha from the PDB file. The threshold for the two protein-contact regions was set at 12 Å rather than 8 Å, which is commonly used [60]. This is because, as a prediction method, the model generated by the AlphaFold Multimer is likely to have a small displacement of atoms in its position. By expanding the general threshold range, more regions can be selected for reference. If all distances in both sequences were greater than the threshold, the threshold was gradually increased until at least one residue was selected for each sequence. The reason for this is to choose an area where the two sequences are spatially closest to each other as the center of the input data; thus, ensuring that the input data provide the greatest possibility for subsequent neural network analysis.

Each tensor is defined to represent a 3D space, and each position in the matrix represents the spatial information in a cube with a side length of 1 Å. The tensor size is restricted to (64, 64, 64, 8). Due to the computing power limitations of the current era, it is practically impossible to continue reducing the per-unit length as it will drastically increase the cost. The first three indexes are coordinates rounded to 1 Å, which means that each tensor stands for a space of cube with the size 64 Å × 64 Å × 64 Å. The last dimension comprises four types of atoms: carbon, nitrogen, oxygen, and sulfur. The four elements located in the two amino acid sequences occupied four positions for a total of eight positions. Hydrogen was excluded because it is not in the main structure predicted by AlphaFold.

After defining the tensor, the next step involved calculating the representation of each atom in the tensor. Three types of tensorization methods were implemented and evaluated: one-hot, volume, and distance encoding.

#### 2.3.1 One-hot encoding

**Fig. 3:**
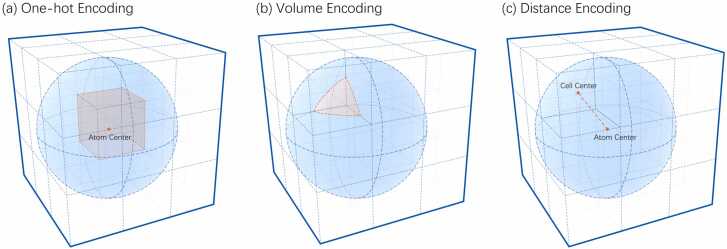
Schematic diagram of the three encoding methods used for rendering atoms into space. The dotted grid represents part of the tensor, while the blue sphere represents the radius of the atom to be calculated. (a) One-hot Encoding: Marks the cell where the atom center is located. The red point in the center represents the atom center, and the cell location is marked in red. (b) Volume Encoding: Values of each cell equal to the volume of the intersection of the cell and the atom. The red part represents the intersection of the closest upper left corner cell and atom, and the volume of this part is marked as the value of the cell. (c) Distance Encoding: Values of each cell are equal to the distance from the atom center to the cell. The red dotted line represents the distance between the closest upper left corner cell and the atom center. This value is marked as the value of the cell.

The most common encoding method is one-hot encoding. This method begins by rounding the coordinates of an atom to an integer to determine its location. The value of that cell was marked as 1 according to the chain and type of atom, whereas all other positions were marked as 0. This method was used by Jones *et al*. [24]. A schematic of one-hot encoding is shown in Fig. 3(a).

An advantage of this method is that it is easy to calculate and understand. The spatial distribution of atoms is expressed directly. Nevertheless, there is also the problem of deleting all the atomic coordinate information after a decimal place. Given that it is generally believed that the condition for the contact of two residues to be considered feasible is that their atomic distance is less than 8 Å; however, the discarded part may be closer to 1 Å. Such large deviations may have harmful effects on the forecast results.

#### 2.3.2 Volume encoding

Volume encoding is a more accurate method to express the spatial distribution of atoms than the one-hot encoding because the atomic radius may not be negligible. The diameters of the atoms in the protein are listed in Table 1. It calculates the volume distribution of atoms and defines the value in each cell as the intersection of the atomic volume and the cell. A schematic diagram of volume encoding is shown in Fig. 3(b). This method can be more accurate for spatial representation than the one-hot method. As the probability that the center of an atom is in the center of a cell is negligible, this method provides a more explicit representation of the spatial distribution of atoms without increasing the input size of the neural network.

**Table 1:** The diameter of the atoms included in PDB files [61]. The atomic diameters of the main atoms constituting amino acids: carbon, nitrogen, oxygen, and sulfur are all larger than the designed unit side length, which is 1 Å.

Atom	Atomic Diameter (Å)
Hydrogen	0.50
Carbon	1.40
Nitrogen	1.30
Oxygen	1.20
Sulfur	2.00

For a cell at coordinates $\left(x_{i},y_{j},z_{k}\right)$, the side length is equal to a, and the center of the atom is defined as $\left(x_{a},y_{a},z_{a}\right)$ with radius R. Let S represent the projection of the intersection between the cell and atom onto the x−y plane. The volume of the intersection can be calculated using the following formula:

$$l=\sqrt{R^{2}-{\left(x_{i}-x_{a}\right)}^{2}-{\left(y_{j}-y_{a}\right)}^{2}}$$

$$\mathrm{volume}=\iint_{S}\left\{\min\left(z_{k}+a,\;z_{a}+l\right)-\;\max\left(z_{k},\;z_{a}-a\right)\right\}d\sigma$$

The implementation of volume calculation for the intersection of the atom and the unit would be calculated through integration. However, this method is time consuming. Therefore, random simulations were used as a replacement. The random simulation method generated 1000,000 random points within the cell. The intersection volume was estimated by counting the number of points inside an atom. The volume calculated using this method was compared with that calculated by integration to verify its effectiveness. The accuracy of this method was approximately 99.5%.

#### 2.3.3 Distance encoding

Distance encoding is based on the characteristics of interatomic interactions. The main factors that affect protein interactions include electrostatic interactions, hydrogen bonds, hydrophobic effects, and van der Waals forces [62]. Distance is the main factor affecting these factors.

The distance encoding algorithm renders the surrounding cells as the distance between the cell and center of the atom, starting from each atomic position. A schematic diagram of distance encoding is shown in Fig. 3(c). One problem with both one-hot and volume encoding is that most of the space is empty when represented because of the nature of the protein complex itself. This property causes the resulting tensor to be treated mathematically as a sparse matrix. Studies have demonstrated that the performance of neural networks deteriorates when the input matrix is too sparse [63]. This is because such a sparse matrix results in each input data point providing updates to only a limited number of neurons, which dramatically reduces the training efficiency. Simultaneously, this sparse matrix causes the trained network to rely more on local details; thus, reducing the network stability. The advantage of distance coding is that it fills most of the data space, making the tensor spatially dense. This method provides relatively rich atomic coordinate information without increasing the amount of data, unlike volume encoding, and improves the training efficiency and quality of the neural network.

Distance is defined as the Euclidean distance from the center of the cell to the center point of the atom. The maximum distance for rendering was set to 12 Å, which is the same as the interface calculation. When a cell is within the threshold of two or more identical atoms simultaneously, the value of the cell is defined as the smaller of the two distances. When a cell was within a threshold of two or more different atoms simultaneously, the distance between the corresponding atoms was marked at the same time. For a cell at coordinates $\left(x_{i},y_{j},z_{k}\right)$, and the center of the atom defined as $\left(x_{a},y_{a},z_{a}\right)$, then the distance can be calculated using the following formula:

$\mathrm{distance}=\sqrt{{\left(x_{i}-x_{a}\right)}^{2}+{\left(y_{j}-y_{a}\right)}^{2}+{\left(z_{k}-z_{a}\right)}^{2}}$

### 2.4 Architecture of neural networks

The number of samples affects model performance. However, due to the specificity of biological data, numerous data augmentation techniques, such as flipping, were inappropriate for this study [64]. Therefore, data augmentation relies on rotations of the input data. Because the input tensor is defined as a cube, the coordinate system can be established from the eight vertices of the cube as the coordinate origins, and interchanging the *x*, *y*, and *z* axes results in 24 rotation possibilities. This approach alleviates data shortcomings. Simultaneously, the network structure was trained to balance by rotation to address the inconsistent orientation of the predicted protein complex. Additionally, random displacement within a ± 6 Å range in the three axial directions have been introduced. By increasing variation in positioning, this approach aims to mitigate the potential for overfitting.

**Fig. 4:**
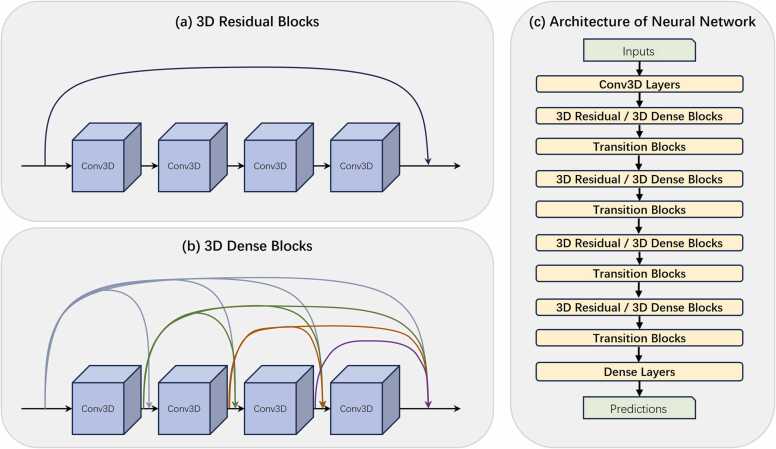
Detailed description of the SpatialPPI network structure. (a) Structure of 3D Residual Blocks. In each residual block, the input data is directly accumulated into the output layer through a shortcut. (b) Structure of 3D Dense Blocks. For dense blocks, the input data and the output data of each layer of the convolutional network except the last layer are passed backward using a shortcut. (c) Architecture of the neural network. Multiple backbone blocks are gradually reduced in size after being connected using transition blocks, and finally, dense layers conclude and output the prediction.

In this study, modified ResNet and DenseNet were used as backbone options for the neural network, with implementations adapted from Ju [65] and Dudovitch [66]. The core architecture of the network consisted of four blocks, each comprising of four convolution 3D layers. ResNet introduces shortcut connections in each block, passing data from the front of a block directly to the back to mitigate the gradient vanishing problem; thereby improving the performance of deep neural networks. DenseNet follows a similar idea by connecting all previous layers to subsequent layers. Following each convolution 3D layer, a dropout layer with a rate of 0.2 is implemented to mitigate the risk of overfitting. The output of each block is processed using a batch normalization layer, followed by an activation layer. Except for the first block, the input to each subsequent block was a reduced output from the preceding block, which was achieved using an average pooling 3D layer. In the final segment of the network, a global max-pooling 3D layer is used to transform the 3D features into a 1D feature vector, which is subsequently fed into a fully connected dense layer, culminating in the prediction of the two classes. A softmax activation function was utilized, which allowed the output to be interpreted as a probability distribution. Fig. 4 shows a diagram of the network architecture. The model consisted of 633,962 trainable parameters. During the training process, the initial learning rate was set to 10^-5^, with a regularization strategy involving weight decay, set to 10^-4^. The order of the training dataset was shuffled at the end of each epoch to enhance the training process diversity. The entire training regimen spanned 40 epochs, with a batch size of 32. Finally, the output of the entire network is a 2-length array, representing the probabilities of the input data being positive and negative. For the loss function, positive data were labeled as [0,1] and negative data as [0,1], whereas the weights were updated by calculating the categorical cross-entropy between the predicted results and labels.

### 2.5 Evaluation methods

To evaluate the model performance, the dataset was randomly segmented into five non-overlapping subsets. During dataset partitioning, clustering of proteins was performed using MMseqs2 ‘easy-cluster’ [67] to ensure that similar proteins always appear within the same subset. Moreover, the five AlphaFold Multimer-predicted models generated for each protein pair were divided into the same subset to ensure that there was no data overlap between different subsets. In this study, the evaluation of the model is based on 5-fold cross-validation. Within each cross-validation fold, four of these subsets constituted the training set, and the resting subset functioned as the test set. Additionally, for each subset, when serving as training data, the five predicted models from the AlphaFold Multimer for each protein pair utilized for training purposes. Conversely, when a subset was designated as test data, only the first model for each protein pair was used for testing. A comprehensive prediction of the dataset was generated by aggregating the predictions made on the test set over five folds. This process ensured a robust and thorough assessment of the predictive capabilities of the model across the entire dataset.

The Rosetta docking score [68] is introduced to evaluate the performance of AlphaFold-predicted models. The Rosetta docking score incorporates several components, including van der Waals forces, electrostatic interactions, hydrogen bonding, solvation energy, and entropy loss upon binding. This score is crucial for identifying the most stable or likely conformation of a protein complex formed when two or more proteins interact. The lower the docking score, the more favorable the predicted interaction is considered to be, implying a higher likelihood of biological relevance and stability of the complex. The Rosetta docking score was calculated using the Release software v3.13.

## 3 Results and discussion

Multiple sets of tests were conducted to evaluate the model performance. These tests encompassed various experiments involving the comparison of different backbone architectures and tensorization methods, a comparative analysis against other state-of-the-art PPI prediction methods, and a completely independent supplementary test dataset that was also used to validate the results. Regarding the evaluation criteria, binary accuracy (ACC), area under curve (AUC), precision, and recall were used for the model evaluation. The model performance was visually represented by generating receiver operating characteristic (ROC) curves.

### 3.1 Evaluations between different backbone and tensorization methods

**Fig. 5:**
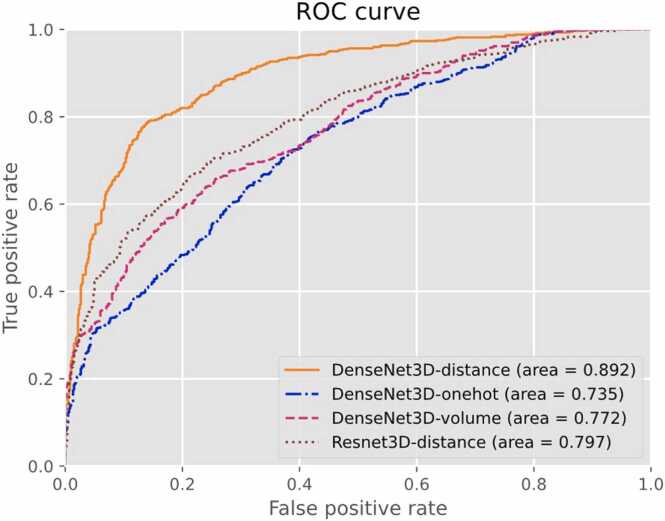
Receiver operating characteristic (ROC) curve for different encoding methods and network backbones based on the 5-fold cross-validation result of the dataset.

SpatialPPI performed 5-fold cross-validation on the dataset using the DenseNet3D backbone with distance, one-hot, and volume encoding, whereas a Resnet3D backbone with distance encoding was also used. The test results are presented in Table 2 and Fig. 5.

**Table 2:** Comparison of accuracy (ACC), area under curve (AUC), precision, and recall for different encoding methods and network backbones based on the 5-fold cross-validation result of the dataset. “Average Deviation” refers to the average deviation of accuracy across five models during the 5-fold cross-validation, while “Standard Deviation” denotes the standard deviation of the accuracy measurements for these models. These metrics provide insights into the overall performance of the models and the consistency of their accuracy.

Backbone	DenseNet3D	DenseNet3D	DenseNet3D	Resnet3D
Tensorization	Distance Encoding	One-Hot Encoding	Volume Encoding	Distance Encoding
ACC	0.818	0.640	0.665	0.714
AUC	0.892	0.735	0.772	0.797
precision	0.832	0.695	0.636	0.788
recall	0.796	0.501	0.771	0.585
True Positive	478	301	463	351
False Negative	122	299	137	249
False Positive	96	132	265	94
True Negative	504	468	335	506
Average Deviation	0.023	0.088	0.061	0.044
Standard Deviation	0.030	0.101	0.078	0.050

The utilization of DensetNet3D as the backbone architecture, along with the distance tensorization method, yielded the best performance across all metrics. Conversely, the performance of the one-hot encoding method was the least satisfactory. This result confirms that distance encoding provides a practical reference for the attention of the neural network to interatomic distances. It also demonstrates spatially dense characteristics, which enable more comprehensive updates of the neural unit weights. Notably, the model using ResNet3D as the backbone architecture had a precision similar to that of the DensetNet3D model. However, the recall of the ResNet3D model might be more satisfactory. This implies that the ResNet3D model can effectively identify true negative data with relatively reasonable accuracy but struggles to detect true positive data. In other words, the structure of the DenseNet3D allows the network to retain some distance information in the input data during transmission, thereby improving its performance. Consequently, the combination of distance encoding and DensetNet3D backbone was selected as the top-performing configuration to represent the performance of SpatialPPI for further analyses and evaluations.

By calculating the proportion of valid values in the input data, we defined the fill rate of the input neural network data as the number of non-zero values in the input tensor divided by the input size. For the three tensorization methods used in this study, the average fill rates in the central area were 3% for one-hot encoding, 20% for volume encoding, and 95% for distance encoding. Other studies have applied Gaussian blurring to one-hot encoding [30]. This method has a fill rate similar to that of volume encoding; however, it lacks the spatial distribution information of atoms that is provided through volume encoding. The distance-based encoding method can fill the spatial sparsity in protein structures, enabling a more comprehensive update of neurons in convolutional neural networks, thereby improving the model performance.

### 3.2 Comparison with the existing PPI prediction method

In this study, four state-of-the-art PPI prediction methods were used as comparison objects to evaluate the performance of the model. These methods include D-script, DeepTrio, PEPPI, and SpeedPPI. Currently, two pre-trained models of D-script are available, the Human D-script model (from the original D-script paper) and the Human Topsy-Turvy model [69] (recommended by developers). Both models were evaluated. Otherwise, the predictive results of DeepTrio were obtained using a 5-fold validation of the dataset.

**Fig. 6:**
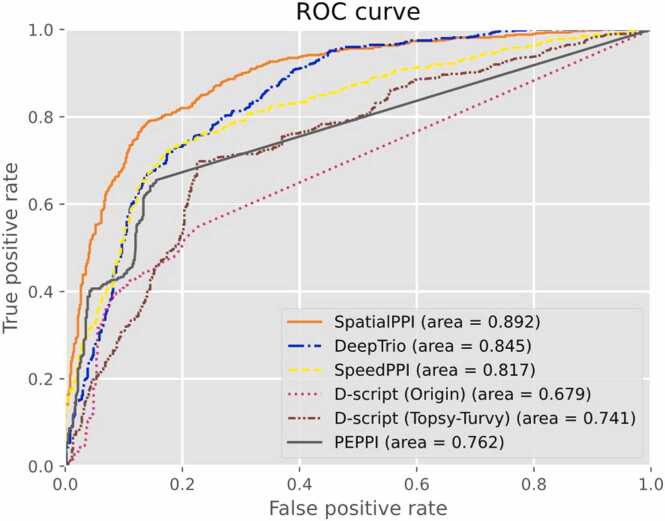
Receiver operating characteristic (ROC) curve for SpatialPPI, DeepTrio, SpeedPPI, PEPPI, and D-script with 2 types of models.

Table 3 and Fig. 6 present the predicted results for each method and SpatialPPI. Overall, SpatialPPI exhibited outstanding performance compared to other similar methods. SpatialPPI achieved the highest accuracy, area under the curve, and recall of all methods. Although PEPPI and the original D-script model display high precision, their ability to detect true positives is less satisfactory. We hypothesize that the training process of the network using conjoint triad features in PEPPI, which shares data sources with the negative dataset in this study, and the inclusion of all proteins in both the negative and positive datasets used in this study may have led to an emphasis on protein sequence characteristics over the analysis of protein-protein associations, resulting in such prediction outcomes. In contrast, the updated D-script Human Topsy-Turvy model exhibits a more balanced performance. DeepTrio performs well overall and boasts the fastest computational speed among all methods. Similar to PEPPI and D-script with human weights, it had a relatively lower but acceptable recall. The protein complexes predicted by the AlphaFold Multimer in the SpatialPPI pipeline were used for the SpeedPPI analysis. The performance of SpeedPPI relies entirely on the pLDDT scores of the predicted interaction residues obtained from AlphaFold by employing a sigmoidal projection. Additionally, SpeedPPI may be influenced since it does not specifically handle the calculation of average pLDDT scores for long single-chain areas that are not structurally entangled with other proteins.

**Table 3:** Comparison of accuracy (ACC), area under curve (AUC), precision, and recall for SpatialPPI, DeepTrio, SpeedPPI, PEPPI, and D-script with 2 types of models.

	**SpatialPPI**	DeepTrio	SpeedPPI	D-script (Origin)	D-script (Topsy-Turvy)	PEPPI
ACC	0.818	0.765	0.773	0.631	0.736	0.680
AUC	0.892	0.845	0.817	0.679	0.741	0.762
precision	0.832	0.830	0.807	0.852	0.755	0.906
recall	0.796	0.667	0.718	0.317	0.698	0.402
True Positive	478	400	431	190	419	241
False Negative	122	200	169	410	181	359
False Positive	96	82	103	33	136	25
True Negative	504	518	497	567	464	575

### 3.3 Evaluations on additional dataset

Experiments were conducted using an additional dataset to validate the network robustness. The additional dataset was a subset of the data utilized in the DeepTrio research [70]. Positive pairs were selected from human species data obtained from the BioGRID database. Negative data were generated by shuffling one sequence from the positive pair. Due to the particularities of amino acid sequences, the probability that shuffled proteins interact with each other is negligible. This method was demonstrated by Kandel *et al.* [71]. Specifically, 571 non-repeating positive pairs and their corresponding shuffled counterparts were selected as the negative dataset. This dataset has a maximum sequences identity of 25% with the standard datasets. Due to the uniqueness of the dataset construction process, SpatialPPI and DeepTrio are evaluated by performing 5-fold cross-validation on this dataset. SpeedPPI, designed without a training process, directly utilizes the same AlphaFold prediction results as used during the SpatialPPI execution. For the execution of SpatialPPI, the DenseNet3D backbone and distance encoding were used. Furthermore, D-script (Topsy-Turvy), was also tested on the additional dataset.

The predicted results are listed in Table 4. The comparison results indicate that SpatialPPI outperforms other methods and demonstrate that the ability of SpatialPPI to learn the spatial structural relationships of residues is also applicable to other datasets. Additionally, a significant difference was observed in the performance on the negative dataset compared to the dataset based on Negatome 2.0. Almost all methods showed a notable improvement in accuracy for the negative dataset. This suggests that the manner in which the negative dataset was generated, while theoretically noninteracting, exhibited substantial differences in sequence characteristics from naturally occurring proteins. Our analysis, which is also supported by studies such as Wei *et al*. [42], indicates that negative datasets derived from actual experimentation often result in lower accuracy of PPI prediction models than those based on theoretical constructs. The ultimate aim of PPI models is to address real-world experimental challenges. The purpose of PPI prediction models is to facilitate the experimental process by filtering protein pairs that are likely to interact and that inherently possess a higher potential for interaction than purely random protein pairs. Therefore, we believe that using real experimental data offers greater reliability than theoretically constructed negative datasets.

**Table 4:** Comparison of accuracy (ACC), area under curve (AUC), precision, and recall for SpatialPPI, DeepTrio, SpeedPPI, and D-script using the Topsy-Turvy model, performed on the additional dataset.

	**SpatialPPI**	DeepTrio	SpeedPPI	D-script (Topsy-Turvy)
acc	0.835	0.833	0.799	0.806
auc	0.920	0.884	0.911	0.879
precision	0.845	0.936	0.925	0.869
recall	0.828	0.714	0.650	0.720

### 3.4 Quality assessment of the AlphaFold Multimer predictions

**Fig. 7:**
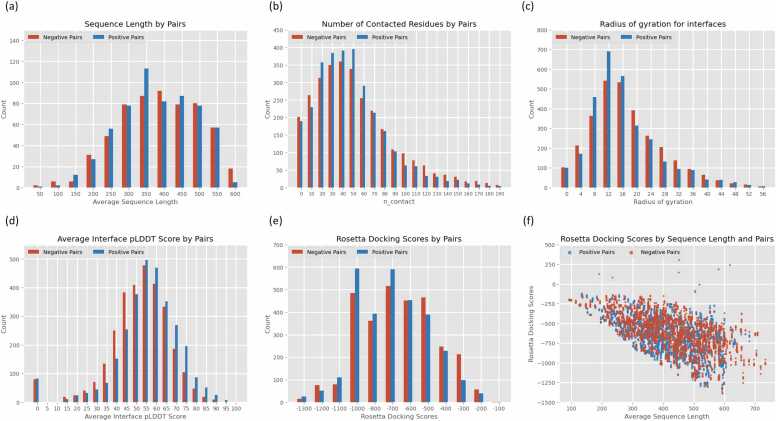
Quality assessment of the AlphaFold Multimer predictions on the dataset. The threshold used to measure residue contacts was 8 Å. (a) Sequence length distribution of negative pairs (Red) and positive pairs (Blue). (b) The distribution of the number of contacted residues of negative pairs (Red) and positive pairs (Blue). (c) The distribution of radius of gyration for the interfaces between negative pairs (Red) and positive pairs (Blue). (d) The distribution of average interface pLDDT scores for negative pairs (Red) and positive pairs (Blue). (e) The distribution of Rosetta docking scores for negative pairs (Red) and positive pairs (Blue). (f) The scatter plot of Rosetta docking scores versus sequence length between negative pairs (Red) and positive pairs (Blue).

To analyze the differences between the positive and negative pairs predicted by the AlphaFold Multimer prediction model, we calculated the sequence length, number of contacted residues, average interface pLDDT scores, and Rosetta docking score [71] of the predicted model. A histogram of the calculation results is presented in Fig. 7. In particular, the Rosetta docking score was designed to predict the strength and stability of PPIs in a predicted complex structure. As illustrated in Fig. 7(d), both negative pairs and positive pairs exhibit a notable portion in terms of the average interface pLDDT score. This deviation forms the foundational basis for SpeedPPI to distinguish true PPIs. Nevertheless, the intersection between negative pairs and positive pairs in their distribution is significantly larger than their difference, making it challenging to differentiate PPIs solely based on intuitive methods or mathematical approaches through the analysis of average interface pLDDT scores. However, in relation to Fig. 7(a), (b), (c), (e), and (f), no significant distinction between latent and positive data is observed. Consequently, such predictive outcomes are challenging to differentiate through heuristic methods. Furthermore, we calculated the RMSD between five different models predicted by the AlphaFold Multimer for the same protein pair, with an average value of 19 Å. This shows that there are distinct structural differences between the multiple models predicted by the AlphaFold Multimer. This helps ensure diversity in the training data.

Although the AlphaFold Multimer achieves an unprecedented high accuracy in predicting protein complex structures, there is no evident difference between the models it outputs when processing positive and negative pairs. By exploiting this feature, we were able to generate a three-dimensional spatial model of the negative protein dataset, thereby providing structural data for the neural network analysis of protein interactions. Alternatively, SpatialPPI can use protein structural information to predict protein interactions, even for proteins whose 3D structures have not been experimentally determined. However, because intrinsically disordered regions in proteins are represented by a ribbon-like appearance, which should be interpreted as a prediction of a disordered state and not as structural information, their spatial contact with another protein should be ignored. By introducing a process that removes disordered areas, SpatialPPI can correctly distinguish protein interactions without interference from entangled disordered areas, which is critical to the classification capabilities of SpatialPPI.

**Fig. 8:**
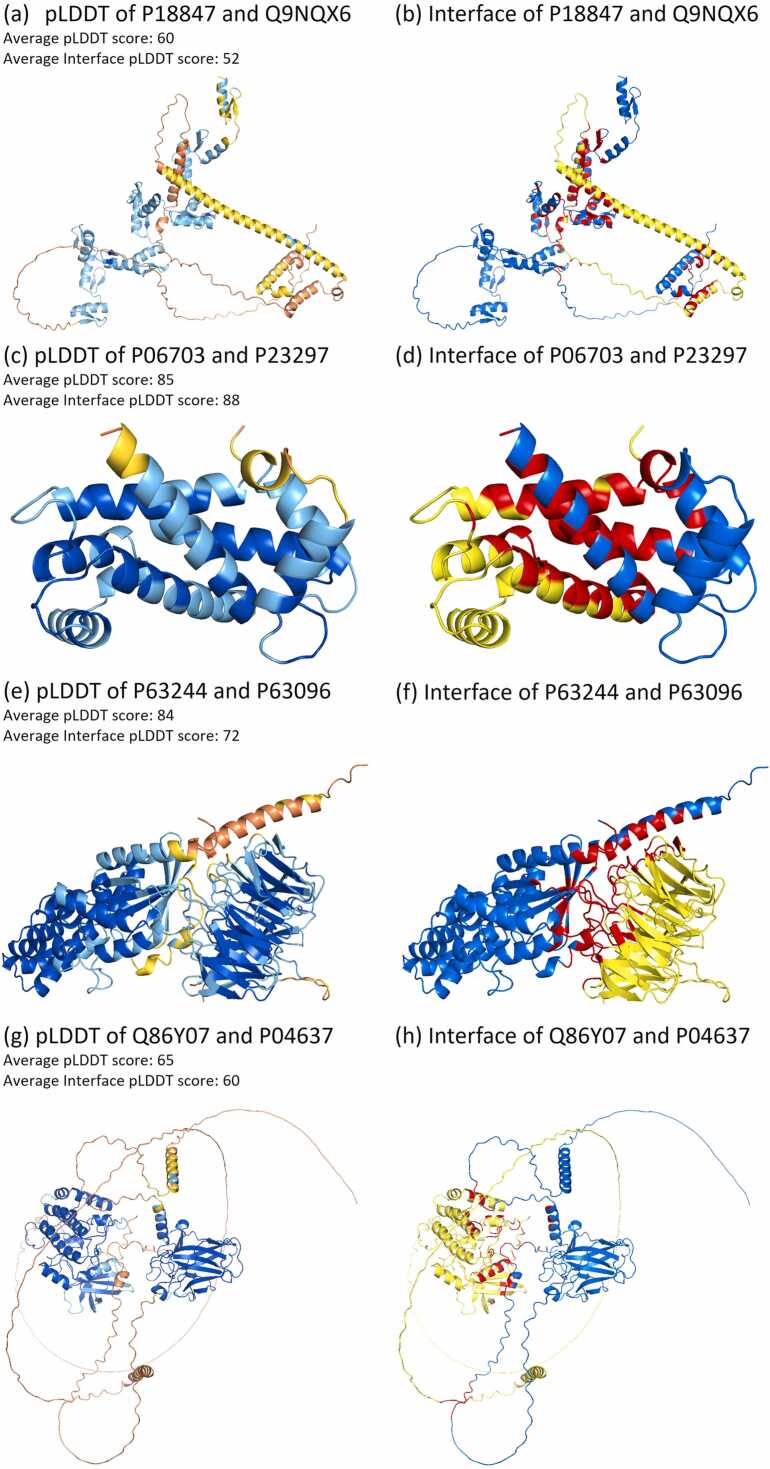
Examples of structures predicted by the AlphaFold Multimer. (a)(c)(e)(g) pLDDT scores of predicted structures, where colors stand for the value of the pLDDT score. Blue: Very high (pLDDT > 90); Cyan-blue: High (90 > pLDDT > 70); Yellow: Low (70 > pLDDT > 50); and Orange: Very low (pLDDT < 50). The average plddt score of each protein complex and their average plddt score of the interface are labeled in the figure. (b)(d)(f)(h) Interface between two chains. The blue and yellow parts represent two chains respectively, and the red area represents interface residues whose distance to each other is less than 8 Å. Among the models, (a, b) P18847-Q9NQX6, (c, d) P06703-P23297, and (e, f) P63244-P63096 are experimentally determined pairs of proteins that cannot interact. (g, h) Experimentally determined interacting pair.

Some specific predicted structures of the AlphaFold Multimer are shown in Fig. 8. These models were correctly classified using SpatialPPI. Fig. 8(a) and (b) show a model containing intertwined disordered areas, which form additional contact areas. Fig. 8(c) and (d) and (e) and (f) show two models with high pLDDT scores, which also have very high pLDDT scores in the interface. All three models were derived from negative protein pairs. The models shown in Fig. 8(g) and (h) were derived from positive protein pairs; however, they were relatively separated in space, and only some disordered areas interacted with each other.

In addition, we verified the performance of the AlphaFold Multimer and SpatialPPI on the protein data from CASP14. The test data were obtained from the FoldDock GitLab [29]. Due to the overlap between the CASP14 dataset and the standard datasets, the model used in this testing was trained on the standard dataset by removing records with sequence identities greater than 40% of the sequences in the CASP14 dataset. Table 5 shows the comparative effectiveness of accurate AlphaFold Multimer predictions and correct SpatialPPI predictions. It is noteworthy that the table categorizes predictions based on the RMSD values, with a threshold of 5 Å serving as a demarcation point.

**Table 5:** Comparison of accurate AlphaFold Multimer prediction and correct SpatialPPI prediction.

	Correct Prediction	False Prediction	Total
RMSD < 5 Å	11	2	13
RMSD > 5 Å	2	2	4
Total	13	4	17

This table illustrates that the predictive capabilities of SpatialPPI are not solely dependent on the proximity of residues, as determined by the AlphaFold Multimer model. Instead, SpatialPPI appears to be more attuned to the spatial data composition characteristics of the proteins. For instance, in cases where RMSD values were less than 5 Å, SpatialPPI accurately predicted protein interactions 11 out of 13 times, indicating its ability to discern subtle spatial nuances. However, even with RMSD values greater than 5 Å, SpatialPPI correctly identified 2 out of 4 cases. This indicates that even AlphaFold predicted models with higher RMSD values can capture essential structural features and correct interactions crucial for PPI prediction. These models may retain significant aspects of the protein's overall shape or key interaction sites, sufficient for accurate PPI identification. And it also states the robustness of SpatialPPI. It underscores the potential to extract valuable insights from imperfect structural models, which remain reliable even in less accurate models.

## 4 Conclusion

In conclusion, SpatialPPI is a pipeline for predicting PPIs that utilizes protein complex structures predicted using the AlphaFold Multimer. By testing and evaluating three strategies for rendering protein structure information into spatial tensors and two commonly used backbones for image recognition and video classification, this study provides a reference for analyzing protein 3D structures using neural networks. Additionally, this highlights that both protein structure data and image data are fundamentally based on spatially distributed information. This commonality in their feature characteristics enables their capture and classification by similar CNN architectures. Furthermore, the necessity of using experimentally validated negative datasets was demonstrated by testing them on two independent datasets. With advancements in computational capabilities, we hope that this analytical approach will further develop and contribute to the understanding of the principles underlying PPIs.

SpatialPPI could enable the detailed elucidation of molecular architectures and the intricate networks they form; thus, facilitating groundbreaking insights into cellular mechanisms. Accurate PPI predictions are pivotal for advancing drug discovery as they allow the identification of novel therapeutic targets and the development of efficacious drugs with fewer off-target effects. Furthermore, these approaches aid in the engineering of proteins with bespoke functions, thereby bolstering innovations in biotechnology and synthetic biology. This innovative methodology not only contributes to the clarification of protein interaction networks but also provides a metric for evaluating predictive models of protein complexes. When addressing unknown protein sequences, SpatialPPI can circumvent model generation using predictive methods that falsely suggest non-interacting sequences. Conversely, if there is a high likelihood of interaction between sequences in a model, the predictive model yields two isolated structures, and SpatialPPI can serve as a benchmark for refining the predictive algorithm. This dual functionality enhances predictive accuracy, ensuring that the resulting models reliably reflect the true interaction potential of protein sequences within a complex.

## CRediT authorship contribution statement

**Wenxing Hu**: Conceptualization, Methodology, Software, Validation, Formal analysis, Investigation, Resources, Data curation, Visualization, Writing – original draft. **Masahito Ohue**: Conceptualization, Methodology, Formal analysis, Investigation, Writing – review & editing, Supervision, Project administration, Funding acquisition.

## Declaration of Competing Interest

The authors declare that they have no known competing financial interests or personal relationships that could have appeared to influence the work reported in this paper.
